# Monitoring der HIV-Präexpositionsprophylaxe in Deutschland auf Basis von GKV-Routinedaten

**DOI:** 10.1007/s00103-025-04165-8

**Published:** 2025-11-27

**Authors:** Frederik Valbert, Daniel Schmidt, Christian Kollan, Martin Friebe, Konstantinos Voulgaris, Barbara Gunsenheimer-Bartmeyer, Jürgen Wasem, Anja Neumann

**Affiliations:** 1 Lehrstuhl für Medizinmanagement, Universität Duisburg-Essen, Essen, Deutschland; 2 Abteilung für Infektionsepidemiologie, Robert Koch-Institut, Berlin, Deutschland; 3 Thea-Leymann-Str. 9, 45127 Essen, Deutschland

**Keywords:** HIV, PrEP, Routinedaten, GKV, Monitoring, HIV, PrEP, Claims data, SHI, Monitoring

## Abstract

**Hintergrund:**

Seit September 2019 ist die HIV-Präexpositionsprophylaxe (PrEP) im Leistungskatalog der Gesetzlichen Krankenversicherung (GKV) enthalten. Ziel des präsentierten Moduls der PrEP-Surv-Studie war ein Monitoring des PrEP-Geschehens in den Jahren 2019 bis 2022 sowie eine Untersuchung der Eignung von GKV-Routinedaten für ein jahrweises Monitoring der PrEP-Versorgung.

**Methoden:**

PrEP-Nutzende wurden in anonymen Daten der BARMER aus den Jahren 2019 bis 2022 identifiziert und analysiert. Gegenstand der Analysen waren jahrweise eine Bestimmung der Anzahl der PrEP-Nutzenden, eine Deskription der Kohorten hinsichtlich Alter, Geschlecht und Therapietreue, eine Identifikation von HIV-Infektionen sowie die Ermittlung der Raten sexuell übertragbarer Infektionen beziehungsweise Infektionskrankheiten (STI).

**Ergebnisse:**

Von 2019 bis 2022 wurde ein Anstieg der PrEP-Nutzendenanzahl von 1058 auf 3194 beobachtet. Demografische Merkmale blieben dabei weitgehend unverändert, während die durchschnittliche zensierte Therapietreue von 98 % im Jahr 2019 auf 91 % bis 92 % in den Folgejahren fiel. Bei 2 Personen wurden HIV-Infektionen beobachtet, wobei die Transmissionen mutmaßlich nicht unter PrEP-Nutzung stattfanden. Hinsichtlich der STI-Raten waren im Zeitverlauf keine klaren Veränderungen zu beobachten.

**Diskussion:**

Die beobachtete Kohorte scheint gut mit anderen PrEP-Kohorten in Deutschland vergleichbar zu sein. Die Stabilität der demografischen Merkmale kann möglicherweise ein Hinweis darauf sein, dass Personengruppen mit PrEP-Indikation, die zu Beginn der Kostenerstattung nicht erreicht wurden, auch weiterhin nicht erreicht werden. Neben einer Deskription des PrEP-Geschehens konnte ein Ansatz aufgezeigt werden, wie ein jährliches Monitoring auf Basis von GKV-Routinedaten umsetzbar wäre.

## Abstract (en)

**Background:**

Since September 2019, HIV pre-exposure prophylaxis (PrEP) has been covered by statutory health insurance (SHI) in Germany. The objectives of the PrEP-Surv study module presented here were to monitor PrEP usage from 2019 to 2022 and to examine the feasibility of using SHI claims data for an annual monitoring of PrEP provision.

**Methods:**

PrEP users were identified and analyzed using anonymized BARMER data from 2019 to 2022. The analyses included determining the number of PrEP users each year; describing the cohorts by age, gender, and treatment adherence; identifying HIV infections; and calculating the rates of sexually transmitted infections or infectious diseases (STIs).

**Results:**

The number of PrEP users increased from 1058 in 2019 to 3194 in 2022. Demographic characteristics remained largely unchanged, while the average censored treatment adherence decreased from 98% in 2019 to 91% and 92% in the subsequent years. Two HIV infections were observed in two individuals, but the transmissions presumably occurred outside of PrEP use. No clear changes in STI rates were observed.

**Discussion:**

The observed cohort appears to be comparable to other PrEP cohorts in Germany. The stability of the demographic characteristics possibly indicates that groups eligible for PrEP that were not reached at the beginning of PrEP reimbursement were subsequently also not reached. In addition to a description of PrEP care, a possible approach for the annual monitoring based on SHI claims data is presented.

## Hintergrund

Die HIV-Präexpositionsprophylaxe (PrEP) gilt als effiziente und kosteneffektive Methode zur Prävention einer Infektion mit dem humanen Immundefizienzvirus (HIV; [1–4]). Seit September 2019 ist die PrEP in Deutschland für Personen mit erhöhtem HIV-Infektionsrisiko im Leistungskatalog der Gesetzlichen Krankenversicherung (GKV) enthalten.

Die Einführung der PrEP als GKV-Leistung wurde im Auftrag des Bundesministeriums für Gesundheit in der „Evaluation der Einführung der HIV-Präexpositionsprophylaxe als Leistung der gesetzlichen Krankenversicherung“ (EvE-PrEP) für die Jahre 2019 und 2020 wissenschaftlich begleitet [5]. Daran anschließend beauftragte das Bundesministerium für Gesundheit die Studie zur „Surveillance der Versorgung mit der HIV-Präexpositionsprophylaxe innerhalb der GKV in Deutschland“ (PrEP-Surv; [6]).

In dem hier präsentierten Modul der PrEP-Surv-Studie wurden GKV-Routinedaten analysiert, sowohl mit dem Ziel eines Monitorings des PrEP-Geschehens in den Jahren 2019 bis 2022 als auch um zu untersuchen, inwiefern GKV-Routinedaten für ein kontinuierliches, jahrweises Monitoring der PrEP-Versorgung geeignet sind.

## Methoden

Als Datengrundlage der Erprobung von GKV-Routinedaten als eine Basis für ein jährliches Monitoring des PrEP-Geschehens bei Menschen mit GKV in Deutschland wurden anonyme Routinedaten im Wissenschafts-Data-Warehouse der BARMER (WDWH) aus den Jahren 2019 bis 2022 ausgewertet. Obwohl im WDWH jahresübergreifende Analysen möglich sind, wurden in dem hier vorgestellten Modul der PrEP-Surv-Studie die Jahre getrennt voneinander betrachtet, um ein jährliches Monitoring anhand von GKV-Routinedaten zu erproben. In einem anderen Modul der Studie wurde der gleiche Zeitraum jahresübergreifend analysiert.

Als PrEP-Nutzende wurden jahrweise in den Daten alle Personen identifiziert, die folgende Merkmale erfüllten:

- mindestens einen ambulanten Behandlungsfall mit mindestens einer PrEP-typischen Abrechnungsziffer im Datenjahr (operationalisiert durch die Ziffern des Einheitlichen Bewertungsmaßstabs der Kassenärztlichen Bundesvereinigung (EBM) 01920, 01921, 01922, 01930, 01931, 01932, 01933, 01934, 01935, 01936),
- mindestens eine alleinstehende^1^ Verordnung der Kombination aus Tenofovirdisoproxil und Emtricitabin (operationalisiert durch den Anatomisch-Therapeutisch-Chemische-Einordnung-von-Wirkstoffen-und-Arzneimitteln-(ATC-)Code J05AR03) zeitgleich oder nach der frühesten PrEP-typischen Abrechnungsziffer im Datenjahr und
- maximal 5 Tage am Stück nicht bei der BARMER versichert (ausgenommen hiervon sind Fehlzeiten vor Versicherungsbeginn oder nach Versicherungsende).

Durch dieses Aufgreifschema sollte sichergestellt werden, dass weder Personen eingeschlossen werden, die zwar eine Beratung zur PrEP bekamen, bei denen es jedoch nie zu einer entsprechenden Medikamentenabgabe kam, noch Personen, die Tenofovirdisoproxil und Emtricitabin als Teil einer (aufgestockten) Postexpositionsprophylaxe oder HIV-Therapie bekamen. Zudem sollte der Versichertenverlauf nur geringfügige Lücken aufweisen, um keine relevanten Outcomes zu verpassen.

Zur Beschreibung der PrEP-Population sollten die Anzahl der Nutzenden, die jeweiligen Geschlechteranteile (administratives Geschlecht) sowie das durchschnittliche und das mediane Alter im Datenjahr deskriptiv berichtet werden.

Das PrEP-Einnahmeschema ist aus Routinedaten nicht direkt ersichtlich. Zur Bestimmung der Therapietreue wurde von einer täglichen Einnahme ausgegangen, was der Empfehlung der aktuellen Leitlinien entspricht, auch wenn ein On-Demand-Use nicht ausgeschlossen werden kann [7]. Entsprechend wurde die Therapietreue als der Quotient aus abgegebenen PrEP-Tagesdosen und angenommenen Tagen mit PrEP-Nutzung berechnet. Die Tage mit angenommener PrEP-Nutzung erstrecken sich vom Datum der ersten Abgabe zeitgleich oder nach der frühesten PrEP-typischen Abrechnungsziffer im Datenjahr bis zum Datum der letzten Abgabe plus der dort abgegebenen Tagesdosen. Wenn Tagesdosen über das Datenjahr hinausgingen, wurden sowohl das PrEP-Ende als auch die Anzahl der berücksichtigten Tagesdosen auf den 31.12. des jeweiligen Datenjahres zensiert. Es war bereits aus der EvE-PrEP-Studie bekannt, dass vereinzelt Personen mit einem Quotienten von über 1 beobachtet werden könnten [8]. Um Verzerrungen der durchschnittlichen Therapietreue hierdurch zu verhindern, wurde zusätzlich die durchschnittliche Therapietreue mit auf maximal 1 zensierten Quotienten berechnet.

Da in der EvE-PrEP-Studie vergleichsweise oft parallel zu PrEP-Abrechnungsziffern augenscheinlich unzutreffende Codes der Internationalen statistischen Klassifikation der Krankheiten und verwandter Gesundheitsprobleme Version 10 (ICD-10) für HIV beobachtet wurden, wurde eine Methodik entwickelt, um zu überprüfen, ob tatsächlich HIV-Fälle unter PrEP auftraten. Diese Methodik geht über das häufig verwendete M2Q-Kriterium (Auftreten der Diagnose in mindestens 2 Kalenderquartalen) hinaus. In einem ersten Schritt wurde ein personenindividuelles Falsifizierungsdatum ermittelt. Dies ist das späteste Datum im Beobachtungsjahr, an dem eine der folgenden Bedingungen zutrifft: Beginn eines Behandlungsfalles, in dem die PrEP-typische EBM-Ziffer 01920, 01921, 01922 oder 01931 abgerechnet wurde, oder Verordnungsdatum einer als PrEP eingestuften Medikation (alleinstehende Verordnung der Kombination aus Tenofovirdisoproxil und Emtricitabin). In einem zweiten Schritt wurde in gesicherten ambulanten Diagnosen, gesicherten krankenhausambulanten Diagnosen, stationären und Arbeitsunfähigkeitsdiagnosen nach den HIV-ICD-10-Codes B20, B21, B22, B23, B24, O89.7 und Z21 gesucht. Dabei wurden nur Diagnosen berücksichtigt, die nach dem individuellen Falsifizierungsdatum begannen. Bei den so identifizierten Personen wurden anschließend die GKV-Routinedaten in Einzelfallprüfungen im Detail in allen verfügbaren Versorgungsdaten (EBM-Ziffern, ICD-10-Codes, ATC-Codes) auf Hinweise für oder gegen eine HIV-Infektion untersucht.

Auch bei der Analyse der Raten sexuell übertragbarer Infektionen beziehungsweise Infektionskrankheiten (STI) wurden gesicherte ambulante, gesicherte krankenhausambulante, stationäre und Arbeitsunfähigkeitsdiagnosen ausgewertet. Die Operationalisierung der hier betrachteten STI ist Tab. 1 zu entnehmen. Berücksichtigt wurden nur Fälle, bei denen der Behandlungsfallbeginn beziehungsweise die Aufnahme zeitgleich oder nach dem PrEP-Beginn und zeitgleich oder vor dem PrEP-Ende lagen. Die aufgegriffenen ICD-10-Codes wurden zu den in Tab. 1 genannten Infektionen vergröbert. Wiesen verschiedene ICD-10-Codes der gleichen Erkrankung bei einer Person identische oder überschneidende Diagnosezeiträume auf, wurden sie zu einem Fall zusammengefasst. Zusätzlich zu der absoluten Anzahl beobachteter Fälle wurden die Fälle pro 100.000 Personenjahre, einschließlich der 95 %-Konfidenzintervalle, berechnet. Die zugrunde gelegten Personenjahre entsprechen der angenommenen Zeit unter PrEP.

**Tab. 1:** ICD-10-Kodierung von sexuell übertragbaren Infektionen beziehungsweise Infektionskrankheiten

Erkrankung	ICD-10-Kodierung (ggf. inkl. Unterpunkte)
Chlamydieninfektion	A55, A56
Gonorrhö	A54
Frühsyphilis	A51
Spätsyphilis	A52
Sonstige Syphilis	A53
Syphilis	A51, A52, A53
Trichomoniasis	A59
Anogenitale Warzen	A63.0
Akute Virushepatitis A	B15
Akute Virushepatitis B	B16
Akute Virushepatitis C	B17.1
Chronische Virushepatitis B	B18.0, B18.1
Chronische Virushepatitis C	B18.2
Sonstige und nicht näher bezeichnete chronische Virushepatitis	B18.8, B18.9

Zur Validierung der in den Routinedaten identifizierten Diagnosen wurde geprüft, ob zeitgleich zum Diagnosebeginn (Beginn des Behandlungsfalles in ambulanten und krankenhausambulanten Daten, stationäres Aufnahmedatum oder Beginn der Arbeitsunfähigkeit) mit einem Toleranzzeitraum von ±7 Tagen oder mit einem Toleranzzeitraum von ±14 Tagen die in Tab. 2 gezeigten Arzneimittel verordnet wurden. Es handelt sich hierbei explizit nicht um eine Analyse der Versorgungssituation bei Vorliegen von STI, sondern lediglich um einen Indikator, um die Ergebnisse hinsichtlich der Anzahl der STI weiter einordnen zu können. Dementsprechend wurde keine sehr spezifische Code-Auswahl getroffen, sondern es wurden stark vereinfacht und ausgesprochen breit ATC-Codes berücksichtigt. Es ging in diesem Fall also um eine näherungsweise Abschätzung der Validität der kodierten Diagnosen und nicht um eine Analyse der STI-Behandlung in der Studienkohorte oder eine Inzidenzabschätzung. Deshalb wurden lediglich exakt zeitgleiche Diagnosen eines Behandlungsfalles mit exakt demselben ICD-Code zusammengefasst. Zeitlich überschneidende Diagnosen einer Person und einer Erkrankung wurden im Unterschied zur STI-Analyse nicht aggregiert.

**Tab. 2:** Arzneimittel zur Validierung von sexuell übertragbaren Infektionen beziehungsweise Infektionskrankheiten

Erkrankung	ATC-Code und Wirkstoffnamen der berücksichtigten Medikamente
Chlamydieninfektion	J01FA10 (Azithromycin), J01AA02 (Doxycyclin), J01MA01 (Ofloxacin), J01FA01 (Erythromycin), J01CA04 (Amoxicillin), J01CR02 (Amoxicillin und Beta-Lactamase-Inhibitoren), J01MA14 (Moxifloxacin), J01DD04 (Ceftriaxon), A01AB17 (Metronidazol), J01CR05 (Piperacillin und Beta-Lactamase-Inhibitoren), S01AA26 (Azithromycin), A01AB22 (Doxycyclin), S01AE01 (Ofloxacin), D10AF02 (Erythromycin), S01AE07 (Moxifloxacin), D06BX01 (Metronidazol), S02AA16 (Ofloxacin), S01AA17 (Erythromycin), G01AF01 (Metronidazol), J01XD01 (Metronidazol), P01AB01 (Metronidazol)
Gonorrhö	A01AB17 (Metronidazol), A01AB22 (Doxycyclin), D06BX01 (Metronidazol), G01AF01 (Metronidazol), J01AA02 (Doxycyclin), J01CR02 (Amoxicillin und Beta-Lactamase-Inhibitoren), J01DD01 (Cefotaxim), J01DD04 (Ceftriaxon), J01DD08 (Cefixim), J01FA10 (Azithromycin), J01MA01 (Ofloxacin), J01MA02 (Ciprofloxacin), J01MA12 (Levofloxacin), J01MA14 (Moxifloxacin), J01XD01 (Metronidazol), P01AB01 (Metronidazol), S01AA26 (Azithromycin), S01AE01 (Ofloxacin), S01AE03 (Ciprofloxacin), S01AE05 (Levofloxacin), S01AE07 (Moxifloxacin), S02AA15 (Ciprofloxacin), S02AA16 (Ofloxacin), S03AA07 (Ciprofloxacin)
Syphilis	A01AB22 (Doxycyclin), D10AF02 (Erythromycin), S01AA17 (Erythromycin), J01**** (Antibiotika zur systemischen Anwendung)
Akute Virushepatitis C	J05AP57 (Glecaprevir und Pibrentasvir), J05AP11 (Grazoprevir), J05AP56 (Sofosbuvir, Velpatasvir und Voxilaprevir), J05AP52 (Dasabuvir, Ombitasvir, Paritaprevir und Ritonavir), J05AP53 (Ombitasvir, Paritaprevir und Ritonavir), J05AP05 (Simeprevir), J05AP02 (Telaprevir), J05AP03 (Boceprevir), J05AP51 (Sofosbuvir und Ledipasvir), J05AP08 (Sofosbuvir), J05AP10 (Elbasvir), J05AP55 (Sofosbuvir und Velpatasvir), J05AP07 (Daclatasvir), J05AP09 (Dasabuvir), J05AP01 (Ribavirin), J05AP54 (Elbasvir und Grazoprevir)

* Einschließlich aller Varianten in den weiteren Stellen

Die Datenaufbereitung und -analyse wurden für alle Datenjahre getrennt voneinander mit RStudio (Version 4.31) mit den Packages dplyr, lubridate, bit64, tidyr und epiR durchgeführt. Da ausschließlich anonyme Daten ausgewertet wurden, von denen nicht auf natürliche Personen geschlossen werden kann, war das Einholen eines Ethikvotums für das Forschungsvorhaben nicht notwendig.

## Ergebnisse

Mit dem in der Methodik beschriebenen Algorithmus konnte ein Monitoring des PrEP-Geschehens bei Versicherten der BARMER für die Jahre 2019 bis 2022 durchgeführt werden.

Im WDWH konnten in den Routinedaten über die Jahre 2019 bis 2022 zwischen 1058 und 3194 Personen mit PrEP-Nutzung beobachtet werden. Wie Tab. 3 zeigt, war ein kontinuierlicher Anstieg der PrEP-Nutzenden im Vergleich zum Vorjahr um 91,9 % im Jahr 2020, um 23,9 % im Jahr 2021 und 26,9 % im Jahr 2022 zu beobachten, während das Durchschnittsalter, das mediane Alter und die jeweiligen relativen Häufigkeiten der Geschlechter weitgehend konstant blieben.

**Tab. 3:** Anzahl und Merkmale von PrEP-Nutzenden im Zeitverlauf

	2019	2020	2021	2022
*Anzahl PrEP-Nutzende*	1058	2030	2516	3194
Anzahl männlich (in %)	1048 (99,05)	2008 (98,92)	2493 (99,09)	3158 (98,87)
Anzahl weiblich (in %)	10 (0,95)	22 (1,08)	23 (0,91)	36 (1,13)
*Durchschnittliches Alter (SD)*	38,73 (10,88)	37,99 (10,65)	37,98 (10,85)	38,44 (10,91)
Alter Median (1. und 3. Quartil)	37 (31, 46)	36 (30, 45)	36 (30, 45)	36 (30, 45)

*PrEP* HIV-Präexpositionsprophylaxe, *SD* Standardabweichung

Die durchschnittliche Therapietreue (ausgedrückt als Quotient aus der Anzahl der verfügbaren PrEP-Tagesdosen und den angenommenen Tagen unter PrEP) überschritt im ersten Jahr der PrEP-Erstattung durch die GKV knapp 100 %. In den Folgejahren lag dieser Wert stabil bei gerundet 93 %. Eine noch deutlichere Kontinuität zeigt sich in der medianen Therapietreue, welche lediglich von 100 % im Jahr 2019 auf 99 % in den Folgejahren fiel. Eine Zensur auf einen individuellen Wert von maximal 100 % führt zu etwas geringeren Mittelwerten, die aber in allen Jahren über 90 % liegen und wie der Mittelwert aus nicht zensierten Werten von 2019 auf 2020 abfallen und dann weitgehend stabil bleiben (Tab. 4).

**Tab. 4:** Therapietreue von PrEP-Nutzenden im Zeitverlauf

Therapietreue in %	2019	2020	2021	2022
Therapietreue MW	100,81	92,50	93,21	92,96
Therapietreue SD	13,91	17,36	16,17	16,95
Therapietreue zensiert MW	97,88	90,74	91,50	91,17
Therapietreue zensiert SD	7,74	15,34	14,22	14,55
Therapietreue Median (1. und 3. Quartil)	100,00 (100,00, 100,00)	99,45 (87,51, 100,00)	99,26 (88,71, 100,00)	99,08 (87,95, 100,00)
Therapietreue Minimum	33,04	16,81	19,74	16,14
Therapietreue Maximum	300,00	197,80	160,54	206,11

*MW* Mittelwert, *SD* Standardabweichung

Hinsichtlich der PrEP-Effektivität wurden 25 Personen als PrEP-Nutzende mit HIV-Infektion in den Routinedaten automatisiert aufgegriffen, wovon jedoch 23 in Einzelfallprüfungen als fehlkodiert bewertet wurden (2019: 2, 2020: 7, 2021: 7, 2022: 7). Bei den verbleibenden 2 Personen lassen die Abrechnungsdaten eine entsprechende Diagnose plausibel erscheinen. Die erste Infektion wurde im Jahr 2020 beobachtet. Hier wurde erst eine PrEP mit 35 Tagesdosen abgegeben und 151 Tage später eine antiretrovirale Therapie mit 30 Tagesdosen verschrieben. 2 Tage später folgte eine EBM-Ziffer für eine HIV-Ribonukleinsäure-Bestimmung und weitere 4 Tage später eine Zusatzpauschale für die Behandlung einer Person mit HIV. Es folgten weitere EMB- und ATC-Codes mit Bezug auf eine HIV-Infektion. Der Behandlungsfall, in dem die HIV-Diagnose kodiert wurde, beginnt etwas vor den hier beschriebenen Leistungen und endet nach den Leistungen, womit sich der Zeitpunkt der HIV-Diagnosestellung durch ICD-10-Codes allein nicht eindeutig zuordnen lassen würde und erst durch die weiteren Abrechnungsdaten genauer eingrenzbar wird. Die zweite HIV-Infektion wurde im Jahr 2022 kurz nach Start der PrEP beobachtet. So finden sich 4 Tage nach der ersten PrEP-Verordnung im Datenjahr eine EBM-Ziffer für einen HIV-Test im Rahmen der PrEP und weitere 6 Tage später eine EMB-Ziffer für eine therapiebedürftige HIV-Infektion und eine Zusatzpauschale für die Behandlung einer Person mit HIV. 3 Tage später erfolgte die Verordnung einer antiretroviralen Therapie mit 30 Tagesdosen. Infolge finden sich wiederkehrend ATC-Codes für eine antiretrovirale Therapie. Auch bei diesem Behandlungsfall ist kein eindeutiges Datum aus den ICD-10-Codes allein zuordenbar, der Zeitraum der Diagnose erstreckt sich hier über etwa 2 Monate.

In beiden Fällen erscheint es naheliegend, dass die Transmission nicht unter PrEP-Nutzung stattfand. Aufgrund des Zeitverzugs zwischen der PrEP-Abgabe und der HIV-Diagnose und des Umstands, dass nur 35 Tagesdosen abgegeben wurden, wären bei der ersten Person naheliegend als alternative Erklärungen denkbar: eine Transmission erst nach Abschluss der täglichen PrEP-Einnahme oder eine unzureichende PrEP-Anwendung im Rahmen eines On-Demand-Schemas. Die zweite Person war mutmaßlich bereits bei der PrEP-Verordnung HIV-positiv und wurde dann in diesem Kontext erstmalig diagnostiziert.

Wie in Tab. 5 ersichtlich ist, sind bei PrEP-Nutzenden hinsichtlich der STI pro 100.000 Personenjahre zwar leichte Schwankungen zwischen den Jahren zu beobachten, die 95 %-Konfidenzintervalle der Raten überlappen sich allerdings weitgehend. Klare Veränderungen sind nicht zu beobachten.

**Tab. 5:** Sexuell übertragbare Infektionen beziehungsweise Infektionskrankheiten bei PrEP-Nutzenden – absolute Fallzahl und Fälle pro 100.000 Personenjahre im Zeitverlauf

	2019		2020		2021		2022	
	Fallzahl	Fälle pro 100.000 PJ (95 %-KI)	Fallzahl	Fälle pro 100.000 PJ (95 %-KI)	Fallzahl	Fälle pro 100.000 PJ (95 %-KI)	Fallzahl	Fälle pro 100.000 PJ (95 %-KI)
Chlamydieninfektion	34	18.054,06 (12.502,96–25.228,74)	182	16.233,48 (13.960,63–18.770,90)	229	16.016,13 (14.008,77–18.230,41)	273	14.910,18 (13.193,78–16.787,86)
Gonorrhö	44	23.364,08 (16.976,37–31.365,18)	248	22.120,35 (19.452,73–25.051,62)	371	25.947,54 (23.374,07–28.726,96)	519	28.345,72 (25.959,19–30.892,66)
Frühsyphilis	12	6372,02 (3292,52–11.130,64)	70	6243,65 (4867,23–7888,47)	92	6434,43 (5187,06–7891,26)	123	6717,77 (5583,12–8015,25)
Spätsyphilis	4	2124,01 (578,72–5438,30)	10	891,95 (427,72–1640,33)	16	1119,03 (639,62–1817,24)	14	764,62 (418,03–1282,91)
Sonstige Syphilis	14	7434,02 (4064,25–12.473,03)	72	6422,04 (5024,85–8087,49)	120	8392,73 (6958,41–10.035,65)	145	7919,32 (6682,81–9318,30)
Syphilis*	29	15.399,05 (10.312,99–22.115,61)	150	13.379,24 (11.323,85–15.699,84)	226	15.806,32 (13.812,59–18.006,98)	274	14.964,79 (13.245,16–16.845,71)
Trichomoniasis	0	0	0	0	0	0	0	0
Anogenitale Warzen	26	13.806,05 (9018,57–20.229,06)	180	16.055,09 (13.795,24–18.579,54)	204	14.267,65 (12.376,83–16.365,64)	244	13.326,31 (11.706,52–15.107,57)
Akute Virushepatitis A	1	531,00 (13,44–2958,55)	1	89,19 (2,26–496,96)	3	209,82 (43,27–613,18)	0	0
Akute Virushepatitis B	0	0	9	802,75 (367,07–1523,88)	12	839,27 (433,66–1466,04)	8	436,93 (188,63–860,92)
Akute Virushepatitis C	1	531,00 (13,44–2958,55)	5	445,97 (144,81–1040,76)	1	69,94 (1,77–389,68)	5	273,08 (88,67–637,28)
Chronische Virushepatitis B	4	2124,01 (578,72–5438,30)	21	1873,09 (1159,47–2863,22)	29	2028,24 (1358,35–2912,90)	33	1802,33 (1240,64–2531,14)
Chronische Virushepatitis C	3	1593,01 (328,52–4655,44)	17	1516,31 (883,31–2427,77)	18	1258,91 (746,11–1989,62)	22	1201,55 (753,01–1819,17)
Sonstige und nicht näher bezeichnete chronische Virushepatitis	1	531,00 (13,44–2958,55)	0	0	0	0	1	54,62 (1,38–304,30)

* Frühsyphilis, Spätsyphilis oder sonstige Syphilis (die Anzahl kann von der Summe der Syphilis-Subgruppen abweichen, da zeitgleiche oder überlappende Fälle zusammengenommen werden), *KI* Konfidenzintervall, *PJ* Personenjahre

Tab. 6 zeigt, welche Anteile der ausgewählten STI-Diagnosen in der beobachteten PrEP-Kohorte mit Arzneimittelverordnungen validiert werden konnten. Dabei wurden, wie in der Methodik beschrieben, verschiedene Zeitfenster zwischen Diagnosebeginn und Verordnung des Arzneimittels toleriert. Es lassen sich klare Unterschiede zwischen den verschiedenen Diagnosen beobachten: So konnten in allen Jahren mehr Gonorrhö- und Chlamydiendiagnosen auf diese Weise validiert werden, als dies bei Syphilis- und Virushepatitis-C-Diagnosen der Fall war. Erwartungsgemäß stieg mit einem größeren Zeitfenster auch die Quote validierter Diagnosen, wobei in den allermeisten Fällen das erste Zeitfenster ±7 Tage zusätzliche Toleranz einen stärkeren Effekt auf die Höhe der Quote hat als das zweite Zeitfenster ±14 Tage.

**Tab. 6:** Anteil der mit Arzneimittelverordnungen validierten Diagnosen in %

Anteil der mit Verordnungen validierten Diagnosen in %	2019	2020	2021	2022
Syphilis (exakt)	29,41	44,23	42,62	44,56
Syphilis (±7 Tage)	38,24	51,92	51,05	58,16
Syphilis (±14 Tage)	38,24	55,13	54,43	63,95
Gonorrhö (exakt)	72,00	69,31	71,50	61,78
Gonorrhö (±7 Tage)	86,00	76,90	80,37	73,47
Gonorrhö (±14 Tage)	86,00	78,62	82,01	75,57
Chlamydien (exakt)	71,43	70,30	74,90	61,30
Chlamydien (±7 Tage)	92,86	82,67	84,71	76,71
Chlamydien (±14 Tage)	95,24	84,16	85,88	79,45
Akute Virushepatitis C (exakt)	0,00	16,67	0,00	0,00
Akute Virushepatitis C (±7 Tage)	0,00	16,67	0,00	0,00
Akute Virushepatitis C (±14 Tage)	0,00	16,67	0,00	0,00

## Diskussion

Bezogen auf die bei der BARMER versicherten Personen liegt die Anzahl der hier ermittelten PrEP-Nutzenden in etwa in dem Bereich, der nach einer Routinedatenanalyse von Valbert et al. sowie einem mathematischen Modell von Schmidt et al. für die Jahre 2019, 2020, 2021 beziehungsweise 2022 zu erwarten war [8–10]. In verschiedenen Publikationen, die Charakteristika von PrEP-Nutzenden in Deutschland ab 2019 berichten, schwankt der Anteil der männlichen Nutzer zwischen 98 % und 100 % [5, 8, 11, 12]. Dies entspricht auch dem hier beobachteten Anteil zwischen 98,87 % und 99,09 %. Das hier beobachtete durchschnittliche beziehungsweise mediane Alter liegt im Vergleich zu den publizierten Nutzendencharakteristika eher im oberen Bereich und sehr nahe an den 37,4 Jahren, die von Valbert et al. in Routinedaten anderer Krankenkassen beobachtet wurden [5, 8, 11, 12]. Insgesamt scheint die bei der BARMER beobachtete Kohorte gut mit anderen PrEP-Kohorten in Deutschland vergleichbar zu sein. Aufgrund dieser Vergleichbarkeit wären die Ergebnisse möglicherweise auf die gesamte GKV-Population in Deutschland hochrechenbar, dies war aber nicht Teil der hier präsentierten Analysen. Während sich die zum Vergleich herangezogenen Publikationen auf 2019 und 2020 fokussierten, konnten hier auch Nutzendencharakteristika für die Jahre 2021 und 2022 berichtet werden. Auffällig ist, dass ein stetiger Anstieg der Anzahl von PrEP-Nutzenden beobachtet werden kann, das durchschnittliche beziehungsweise mediane Alter und die Geschlechterverteilung aber über die Jahre weitgehend stabil bleiben. Dies kann möglicherweise ein Hinweis darauf sein, dass Personengruppen mit PrEP-Indikation, die zu Beginn der Kostenerstattung nicht erreicht werden konnten, auch weiterhin nicht erreicht werden. Demzufolge könnten die in anderen Untersuchungen beschriebenen Zugangsbarrieren unverändert bestehen [5, 6].

Hürden bei der Analyse der Adhärenz auf Basis von GKV-Routinedaten liegen in den Umständen, dass das tatsächliche Einnahmeschema und das exakte Ende der PrEP-Nutzung unbekannt sind. Auch wenn in der entsprechenden Leitlinie primär eine tägliche PrEP-Einnahme vorgesehen ist, berichten Schmidt et al., dass der Anteil der PrEP-Nutzenden mit täglicher PrEP in den Studien NEPOS, PrApp und BRAHMS bei lediglich 81 % bis 83 % der PrEP-Nutzenden lag [5]. In Befragungen bei HIV-Schwerpunktzentren im Rahmen von PrEP-Surv war der Anteil mit täglicher PrEP im Jahr 2022 laut Aussage der Zentren mit 67 % noch etwas niedriger [13]. Ein wesentlicher Vorteil der Datenquelle besteht darin, dass GKV-getragene PrEP-Abgaben direkt beobachtet werden konnten und somit keine Verzerrung durch sozial erwünschte Antworten zu erwarten ist. Insgesamt kann vorliegend eine hohe Therapietreue angenommen werden, die über jener liegt, die aufgrund von On-Demand-Use zu erwarten wäre. Auffallend ist auch hier, dass bei der Therapietreue zwischen 2020 und 2022 kaum Veränderungen zu verzeichnen sind. Der Abfall, der sich von 2019 zu 2020 zeigt, ist mutmaßlich vor allem methodisch bedingt: Dadurch, dass die PrEP 2019 erst im September erstattungsfähig wurde, konnten individuell maximal 4 Monate PrEP-Nutzung beobachtet werden. Da nur Personen selektiert wurden, die in dem kurzen Zeitraum mindestens eine PrEP-Abgabe aufwiesen, kommt es hier zu eher hohen Quotienten. Für Quotienten > 1 erscheinen verschiedene Erklärungen denkbar. So kann es beispielsweise sein, dass vorzeitige Verschreibungen eingefordert wurden, weil Tabletten verloren gegangen sind oder vereinzelt andere Personen (ohne entsprechende medizinische Einbettung) mitversorgt wurden. Hier zeigt sich eine Stärke der Routinedatenanalyse. Aus Gründen der sozialen Erwünschtheit werden in Primärdatenerhebungen Quotienten größer 1 vermutlich untererfasst.

Eine generell geringe Anzahl von HIV-Infektionen und der Umstand, dass die beobachteten Infektionen wahrscheinlich nicht unter PrEP-Nutzung stattfanden, zeigen die hohe Effektivität der PrEP, erscheinen mit Blick auf die Literatur plausibel und unterstreichen die Wichtigkeit des HIV-Screenings im Rahmen der PrEP-Verordnung [1–3, 5, 8]. Hinsichtlich des Vorhabens eines jährlichen Monitorings auf Basis von GKV-Routinedaten bleiben aber an dieser Stelle 2 Herausforderungen: Zum einen erschwert es die jahrweise Betrachtung, eine HIV-Fehldiagnose am Jahresende zu widerlegen oder wenigstens einzuordnen, da hierfür relevante Ereignisse zur Bestätigung oder Falsifizierung eventuell erst im nächsten Kalenderjahr auftreten. Zum anderen stellen die beobachteten Fehlkodierungen eine Herausforderung für eine vollständig automatisierte Erhebung dar, die gleichzeitig eine ausreichende Sensitivität und Spezifität gewährleisten soll. Mit Blick auf die zeitliche Einordnung der HIV-Diagnose zeigte sich, dass durch ICD-10-Codes allein keine eindeutige Zuordnung möglich war und erst durch die weiteren Abrechnungsdaten eine genauere zeitliche Eingrenzung möglich wurde.

Bei den hier präsentierten STI-Fallzahlen ist hinsichtlich der Datenbasis und der genutzten Methodik eher eine Überschätzung zu erwarten, da in den Routinedaten nicht sicher erkennbar ist, ob bei jeweils einer Person verschiedene Behandlungsfälle mit entsprechender Diagnose zu einer einzelnen Infektion gehören oder ob es sich um verschiedene Infektionen mit dem gleichen Erreger handelt. Zudem erscheint es denkbar, dass teilweise Diagnosen weitergeführt werden, auch wenn die Erkrankung bereits abgeklungen ist. Diese *a priori* erwartete Verzerrung zeigt sich allerdings nicht durchgehend im Abgleich mit der Literatur. Während die Chlamydienraten in der Routinedatenanalyse in der EvE-PrEP-Studie den hier beobachteten Raten in den Jahren 2019 und 2020 stark ähneln, wurden aus der NEPOS-Studie etwas höhere Chlamydienraten berichtet [5, 8, 14]. Umgekehrt stellt es sich bei der Gonorrhö dar. Hier wurden in der NEPOS-Studie fast identische Raten berichtet, während in der Routinedatenanalyse der EvE-PrEP-Studie höhere Raten beobachtet wurden [5, 8, 14]. Bei den entsprechenden Publikationen wurde nicht nach Früh‑, Spät- und sonstiger Syphilis unterschieden, wodurch nur ein Vergleich auf Ebene der kumulierten Syphilisformen möglich ist. In der NEPOS-Erhebung wurde eine etwas geringere Syphilisrate beobachtet als in der vorliegenden Untersuchung, während in dem Routinedatenmodul der EvE-PrEP-Studie eine sehr ähnliche Rate beobachtet wurde [5, 8, 14]. Übereinstimmend ist aber die Beobachtung, dass die berichteten Syphilisraten verglichen mit den Chlamydien- und Gonorrhöraten tendenziell geringer sind [5, 8, 14]. Sowohl in der präsentierten Analyse als auch in der NEPOS-Studie war die absolute Fallzahl der Virushepatitis-B- und Virushepatitis-C-Infektionen niedrig, was zu Inzidenzraten mit entsprechend breiten Konfidenzintervallen führte [14]. Ein Vergleich mit internationaler Literatur wird als weniger zielführend als der Vergleich mit Ergebnissen aus deutschen Studien betrachtet, da die STI-Raten innerhalb einer PrEP-Population in Wechselwirkung zur allgemeinen Prävalenz dieser Infektionen in der entsprechenden Region stehen. Beim Vergleich der verschiedenen Jahre innerhalb der hier präsentierten Ergebnisse ist kein deutlicher Unterschied zwischen den Jahren zu erkennen und somit auch kein herausstechender Effekt der Maßnahmen zur Kontaktbeschränkung während der COVID-19-Pandemie.

Der Anteil der Diagnosen, die mit Arzneimittelverordnungen validiert werden konnten, schwankt stark zwischen einem Minimum von etwa 29 % (Syphilis, 2019, ohne zeitliche Toleranz) und einem Maximum von etwa 95 % (Chlamydien, 2019, ±14 Tage). Dass ein größerer Toleranzzeitraum zu einem höheren Anteil der validierten Diagnosen führt erscheint plausibel, wobei der größere Effekt beim ersten Zeitfenster von ±7 Tagen zu verzeichnen war. Die Unterschiede zwischen den Infektionen können innerhalb dieser Studie nicht abschließend geklärt werden. Auffällig sind vor allem die eher niedrigen Anteile bei Syphilisdiagnosen, insbesondere, da hier bereits sehr unspezifisch ATC-Codes berücksichtigt wurden. Die Anteile könnten potenziell sowohl durch echte Infektionen ohne beobachtete medikamentöse Therapie als auch durch Fehlkodierungen oder einer fortgeführten Kodierung alter Diagnosen erklärt werden. Ein fortgesetztes Kodieren von Diagnosen erscheint insbesondere bei Syphilis plausibel, da Diagnostik und Therapie im Vergleich zu Chlamydien und Gonorrhö häufig aufwendiger sind und die sogenannte Serumnarbe zu weiteren Kodierungen führen kann.

Als allgemeine Limitationen von GKV-Routinedatenanalysen ist zu erwähnen, dass die Datengrundlage primär zur Abrechnung, nicht für wissenschaftliche Analysen generiert wurde und dass sie anfällig für Fehlkodierungen ist [15, 16]. Auf der anderen Seite weisen GKV-Routinedaten den Vorteil auf, dass keine separate Datenerhebung notwendig ist und dass es sich um Real-World-Daten handelt, womit sie als etablierte Quelle für wissenschaftliche Analysen gelten [17, 18].

## Fazit

Es konnte ein Ansatz aufgezeigt werden, wie ein jährliches Monitoring der HIV-Präexpositionsprophylaxe (PrEP) auf Basis von Routinedaten der Gesetzlichen Krankenversicherung (GKV) (beispielsweise über das Forschungsdatenzentrum Gesundheit am Bundesinstitut für Arzneimittel und Medizinprodukte) kostengünstig umsetzbar wäre. Dabei wurden für die Jahre 2019 und 2020 Ergebnisse generiert, die sich gut in publizierte Evidenz, teils aus Primärdaten, einordnen lassen. Zusätzlich dazu konnte das PrEP-Geschehen bei Versicherten einer der größten gesetzlichen Krankenversicherungen Deutschlands für die Jahre 2021 und 2022 berichtet werden. Über die 4 Jahre war ein stetiger Anstieg der Anzahl der PrEP-Nutzenden zu beobachten. Charakteristika der PrEP-Nutzenden blieben unverändert, was möglicherweise darauf hindeutet, dass die in anderen Studien beschriebenen Zugangsbarrieren weiterhin bestehen. In dieser Untersuchung blieben die Raten sexuell übertragbarer Infektionen beziehungsweise Infektionskrankheiten (STI) unter PrEP weitgehend unverändert, sodass kein Effekt der Kontaktbeschränkungsmaßnahmen im Rahmen der COVID-19-Pandemie beobachtet werden konnte. Zudem zeigte sich die PrEP in den analysierten Jahren als sehr effektiv, bei beiden beobachteten HIV-Infektionen wird von einer Transmission außerhalb der PrEP-Nutzung ausgegangen.

## Footnotes

1 Wenn weder zeitgleich zur Verordnung von Tenofovirdisoproxil und Emtricitabin ein anderes Medikament zur antiretroviralen Therapie verordnet wurde noch eine vorhergehende Abgabe einer anderen antiretroviralen Medikation aufgrund der abgegebenen Tagesdosen die Abgabe von Tenofovirdisoproxil und Emtricitabin überlappt, wurde diese Verordnung als „alleinstehend“ bewertet. Abweichend von der ansonsten jahrweisen Betrachtung wurden bei der Prüfung von überlappenden antiretroviralen Therapien ab dem Jahr 2020 auch Arzneimittel aus dem Vorjahr berücksichtigt, wenn sie aufgrund des Abgabedatums und den abgegebenen Tagesdosen in das jeweilige Datenjahr hineinragten.
